# Sialoglycoproteins and N-Glycans from Secreted Exosomes of Ovarian Carcinoma Cells

**DOI:** 10.1371/journal.pone.0078631

**Published:** 2013-10-24

**Authors:** Cristina Escrevente, Nicolas Grammel, Sebastian Kandzia, Johannes Zeiser, Erin M. Tranfield, Harald S. Conradt, Júlia Costa

**Affiliations:** 1 Laboratory of Glycobiology, Instituto de Tecnologia Química e Biológica, Universidade Nova de Lisboa, Oeiras, Portugal; 2 GlycoThera GmbH, Hannover, Germany; 3 Electron Microscopy Facility, Instituto Gulbenkian de Ciência, Oeiras, Portugal; Institute of Cancerology Gustave Roussy, France

## Abstract

Exosomes consist of vesicles that are secreted by several human cells, including tumor cells and neurons, and they are found in several biological fluids. Exosomes have characteristic protein and lipid composition, however, the results concerning glycoprotein composition and glycosylation are scarce. Here, protein glycosylation of exosomes from ovarian carcinoma SKOV3 cells has been studied by lectin blotting, NP-HPLC analysis of 2-aminobenzamide labeled glycans and mass spectrometry. An abundant sialoglycoprotein was found enriched in exosomes and it was identified by peptide mass fingerprinting and immunoblot as the galectin-3-binding protein (LGALS3BP). Exosomes were found to contain predominantly complex glycans of the di-, tri-, and tetraantennary type with or without proximal fucose and also high mannose glycans. Diantennary glycans containing bisecting N-acetylglucosamine were also detected. This work provides detailed information about glycoprotein and N-glycan composition of exosomes from ovarian cancer cells, furthermore it opens novel perspectives to further explore the functional role of glycans in the biology of exosomes.

## Introduction

Exosomes are membrane vesicles between 40-100 nm in diameter that are secreted by various cell types, including tumor cells, neurons, T-cells, dendritic cells and macrophages. In addition, exosomes were also found in physiological fluids including plasma, cerebrospinal fluid, urine and malignant ascites [1,2]. 

Exosome biogenesis involves the inward budding of endosomes into multivesicular bodies to form intraluminal vesicles that are then released to the extracellular space. The molecular basis of protein sorting during exosomes formation involves a ubiquitin-dependent mechanism but oligomerization or partitioning of protein into lipid raft domains may be involved in the sorting of certain proteins [2]. Exosomes are secreted from the cell by fusion of multivesicular bodies with the plasma membrane. 

Among other functions, exosomes were found to be associated with the transmission of pathogenicity among cells, for example, in tumor progression or neurodegenerative diseases, and they stimulate the immune system [3,4]. Exosomes can interact with target cells through ligand-receptor interactions or lipids, and they have been described to fuse with the plasma membrane or to be internalized via receptor-mediated endocytosis [5,6]. Lectins and their interactions with carbohydrates have also been found to play a role in exosome recognition and uptake by dendritic cells [7] and macrophages [8].

Exosomes have a unique protein, lipid and glycan composition. They contain proteins involved in cellular events including, membrane transport and fusion, biogenesis of multivesicular bodies, tetraspanins, and they are enriched in lipid microdomains [2]. Concerning glycosylation, exosomes were found to contain a conserved glycan signature relatively to parent cell membranes by lectin array technology [9,10]. Furthermore, specific sialic acid-containing glycoproteins were previously found in exosomes from SKOV3 cells [6]. 

In the present work, we have characterized glycoproteins and N-glycans from ovarian carcinoma exosomes. The sialoglycoprotein galectin-3-binding protein (LGALS3BP) was found to be strongly enriched in exosomes. Furthermore, exosomes had complex and high mannose glycans. 

## Materials and Methods

### Cell culture

Human ovarian cancer SKOV3 [11] and OVM [12] cell lines were a generous gift from Prof. Peter Altevogt. Cells were grown in Dulbecco's Modified Eagle Medium high glucose (Sigma), supplemented with 10% fetal calf serum, 100 units/ml penicillin and 0.1 mg/ml streptomycin (Invitrogen), at 37 °C, in 5% CO_2_, as previously described [13]. 

### Separation of exosomes, microsomal and plasma membrane fractions

Cellular extracts were obtained by solubilization of centrifuged cells in 50 mM Tris-HCl pH 7.5, containing 5 mM ethylenediamine tetraacetic acid, 1% Triton X-100, 0.02% protease inhibitors cocktail, Complete, Roche, for 30 min. 

Confluent cells were cultivated for 48 h in serum-free medium. The supernatant was collected and centrifuged at 500, 10,000 and 100,000 x g, 10, 20 and 120 min, respectively, at 4 °C. The pellet of the last centrifugation consisted of crude exosomes, which was further used for exosome purification by sucrose gradient as previously described [5,6]. Essentially, crude exosomes (1 ml suspension) were loaded on top of a stepwise gradient comprising layers of 2 M (1.5 ml), 1.3 M (2.5 ml), 1.16 M (2.5 ml), 0.8 M (2 ml), 0.5 M (2 ml) and 0.25 M (1 ml) sucrose. The gradients were centrifuged for 2.5 h at 100,000x*g* in a Beckman SW41Ti rotor. Twelve 1 ml fractions were collected from the top (1 to 12) and precipitated with 10 % trichloroacetic acid before immunoblotting or lectin blotting analysis.

The isolation of the microsomal fraction was performed by differential centrifugation. Cells were resuspended in homogenization buffer (1 mM NaHCO_3_, 0.2 mM CaCl_2_, 0.2 mM MgCl_2_, 1mM spermidine, pH 8.0) [14], disrupted with a pellet pestle (Sigma) and centrifuged at 600 x g, 10 min, and 15,000 x g, 10 min. The supernatant was further centrifuged at 100,000 x g, for 120 min, and the pellet consisted of the microsomal fraction. 

The isolation of plasma membrane proteins was performed by cell surface biotinylation as previously described [15]. Typically, the following amounts of protein were obtained from cells in one T175: 2 mg for total cell extracts, 0.4 mg for the microsomal fraction, 64 µg for plasma membrane, 36 µg for crude exosomes and 12 µg for exosomes. 

Protein concentration was determined by the bicinchoninic acid method.

### Lectin blotting, immunoblotting and lectin-affinity chromatography

Proteins were analysed by SDS-PAGE in 10% acrylamide gels. Glycoproteins were detected by lectin blotting after transfer to polyvinylidene fluoride as previously described [13]. Concanavalin A (Con A; Sigma), biotinylated Sambucus nigra agglutinin (SNA) and *Maackia amurensis* lectin (MAL) (Galab Technologies) were used. As control of Con A specificity, incubation was done in the presence of competitive sugar, 0.2 M methyl-α-D-mannopyranoside. As controls for SNA and MAL specificity exosomes were incubated with sialidases from *Vibrio cholerae*, *Arthrobacter urefaciens* and *Streptococcus pneumoniae* as previously described [6]. Immunoblotting was done as previously described [6] with goat anti-human GALS3BP polyclonal antibody (R&D) and anti-goat immunoglobulin G coupled to horseradish peroxidase (Sigma) as primary and secondary antibodies at 1:2000 and 1:20000 dilutions, respectively. Detection was performed with the Immobilon Western chemiluminescent (Millipore).

For the separation of MAL-binding proteins, SKOV3 crude exosomes were solubilized in adsorption buffer (Tris-HCl pH 6.0, 1% 3-[(3-cholamidopropyl)dimethylammonio]-1-propanesulfonate) and applied on AffiSpin columns (GALAB Technologies). After several washes with adsorption buffer, MAL-binding proteins were eluted from the column with sample buffer and analysed by SDS-PAGE. 

### Electron microscopy

Preparation of the exosomes for transmission electron microscopy was done using the technique published by Théry et al. [16]. Briefly, centrifuged exosomes were fixed in 2% paraformaldehyde in PBS overnight. Exosomes on 200 mesh carbon coated grids (Ted Pella 01800-F) were further fixed with 1% glutaraldehyde in PBS before being contrasted with uranyl-oxalate and methy cellulose-UA. Grids were observed at 100 kV on a Hitachi-7650 Transmission Electron Microscope and photographed using an AMT XR41-M Mid-mount camera. 

### Peptide mass fingerprinting

Gel bands were excised and subjected to in-gel digestion with trypsin according to the protocol previously described [17]. Tryptic digests of individual gel spots were analyzed by capillary liquid chromatography-tandem mass spectrometry. Liquid chromatography was performed using an Ultimate 3000 RSLCnano. Samples were desalted and preconcentrated at a flow rate of 10 µL/ min on a short C18 Nano Trap Column Acclaim PepMap (5 µm, 100 A, 100 µm i.d. x 2 cm; LC-Packings) connected in the front of an analytical capillary column. For the separation with the C18 PepMap column (180 µm i.d. x 15 cm; LC-Packings), a flow rate of 6 µL/min was used. For the chromatography, the following solvents were used: solvent A (H_2_O, 0.1% formic acid) and solvent B (90% acetonitrile, 9.9% H_2_O, 0.1% formic acid). The eluting gradients used for separation of tryptic digestions were, respectively, 1 to 52% B in 103 min, 52 to 99% B in 1 min, followed by 99% B for 10 min and then by 1% B in 5 min. The chromatography system was directly coupled to a three-dimensional high-capacity ion trap (HCTultra ETD II; Bruker Daltonik, Bremen, Germany) mass spectrometer with an electrospray ionization source. Instruments were controlled using DCMS Link (Dionex) and Compass 1.3 (Bruker Daltonics) software. The smart parameter settings (SPS) were for the target mass 600 m/z and for compound stability 100%. The full MS scan mode was standard-enhanced (m/z 300 to 1600). The two most abundant ions detected in each MS scan were selected for collision-induced dissociation (MS/MS) with 1.0 V collision energy. The peptides were analyzed using the data-dependent MS/MS mode over the m/z range 100–3000. Raw spectrum data were processed and Mascot compatible mgf files were created using DataAnalysis 4.1 software (Bruker Daltonik) with the following parameters: compounds threshold 100,000, maximum number of compounds 300, and retention time windows 0.5 min. Searches were performed using BioTools 3.2 software (Bruker Daltonik) together with the algorithm on the Mascot web site (Matrixscience, London, UK) by searching against the NCBI non-redundant human database. Search parameters were set as follows: enzyme, trypsin; allowance of up to one missed cleavage peptide; mass tolerance, 0.3 Da and MS/MS mass tolerance, 0.3 Da; fixed modification parameter, carbamidomethylation (C). 

### N-glycans isolation and analysis of 2-aminobenzamide labeled N-glycans by NP-HPLC

Total N-glycans isolation from exosome glycoproteins were performed as previously described [18]. 

N-glycans were labelled with 2-AB (2-aminobenzamide) according to the derivatisation method of Bigge [19] with reagents from Sigma-Aldrich. After derivatisation, the 2-AB labelled glycan mixtures were purified from excess labelling reagents by gel filtration using PD MiniTrap G-10 size exclusion columns from GE Healthcare. The size exclusion columns were conditioned with 8 ml ultrapure water prior to sample application. The columns were washed with 700 μl ultrapure water followed by elution of 2-AB labelled N-glycans with 500 μl ultrapure water. 

Mapping of 2-AB-labelled glycans was done with an ACQUITY UPLC BEH glycan column from Waters Corporation (Milford, MA, USA). An HPLC system from Dionex Corporation (Sunnyvale, CA, USA) with an Ultimate 3000 RS Fluorescence detector FLD-3400RS was used. Mobile phase A was 85 % (v/v) acetonitrile and mobile phase B was 250 mM ammonium formate pH 4.4, at a flow rate of 0.40 mL/min and at a column temperature of 60°C. Detection was at excitation wavelength = 347 nm, emission wavelength = 420 nm. The gradient was as follows: starting conditions 88% A and 12% B; a gradient from 1-25 min to 82% A; gradient for 20 min to 70% A; gradient for 10 min to 20% A; increase to 88.0 % A and re-equilibration of the column isocratically for 10 min. Within an analytical sample sequence reference standards were run with various structurally well characterised 2-AB-labelled oligosaccharides. 

Enzymatic digestions of 2-AB-labelled N-glycans with various exoglycosidases were performed with 20-50 pmol of total N-glycans in 10-20 μl of 0.1 M ammonium acetate buffer pH 5.0. Digestions with β(1–4)-galactosidase (bovine testes, Prozyme), α-L-(1–4,6)-fucosidase (bovine kidney, Sigma), α-(1–3,6)-mannosidase digestion (Jack Bean, Sigma) and neuraminidase (*Arthrobacter ureafaciens*, Roche) were performed for 4, 2, 4 and 2 hours, respectively. The glycan incubation mixtures were diluted with 50 μl H_2_O and were applied onto a pipette tip filled with 20-25 μl of porous graphitic carbon (Hypercarb) which had previously been conditioned with 100 μL 80% acetonitrile containing 0.1% trifluoroacetic acid and 150 μl water. After washing with 3 x 150μL H_2_O, N-glycans were eluted with 150 μl 25% acetonitrile and the eluate was dried in a Speed Vac concentrator. Samples were stored at -20°C and were solubilised in 75% acetonitrile / 20% H_2_O and 2-10 pmol of the glycan mixture was used for NP-HPLC glycan mapping. Calculations of peak percentages were done using the Chromeleon 6.8 software.

N-glycan standards were purchased from Thera-Proteins Lda (Portugal): di-, tri- (2,4 and 2,6-branched) and fully α2,3 sialylated tetrantennary (with and without repeats) oligosaccharides with proximal α1-6 linked fucose (Fuc), oligomannosidic standards Man_3-9_GlcNAc_2_, Man_3_GlcNAc_2_ with proximal fucose; other proximal fucosylated standards were diantennary structures without 1 Gal (both, Man-3 and Man-6 branch), diantennary without 2 galactose (Gal), diantennary without 2 Gal and without 1 N-acetylglucosamine (GlcNAc) (both, Man-3 and Man-6 branch); hybrid-type structures with 5 and 6 mannose (Man) residues with and without proximal Fuc. Diantennary with bisecting GlcNAc and proximal Fuc and variants lacking one or two Gal were isolated from human immunoglobulin G. Structures were confirmed by mass spectrometry and NMR.

### Analysis of N-glycans by MALDI-TOF MS

Glycans were analysed with a Bruker ULTRAFLEX time-of-flight (TOF/TOF) instrument. Mass spectra were recorded in the reflector and positive ion mode. Samples of 1 μl at an approximate concentration of 5 pmol.μl-1 were mixed with equal amounts of the matrix 2,5-dihydroxybenzoic acid. The mixture was spotted onto a stainless steel target and dried at room temperature before analysis. Structures were tentatively assigned to various N-glycans on the basis of their observed masses in comparison to the calculated masses of known N-glycans. Further annotation of N-glycans and database search were performed using the Glyco-Peakfinder platform [20]. Further annotation of N-glycans and database searches were performed using the GlycoWorkbench software [21].

### Analysis of 2-AB labeled oligosaccharides by LC-ESI-TRAP-MS

The 2-AB labeled oligosaccharide mixture was analyzed by capillary liquid chromatography-tandem mass spectrometry on the same system used for the peptide analysis but with different parameter settings as follows. Samples were directly injected onto a C18 PepMap column (180 µm i.d. x 15 cm; LC-Packings) at a flow rate of 8 µL/min. The gradient sequence used for chromatographic separation was: 0% B for 5 min; 0% to 35% B in 15 min; 35% to 95% B in 5 min, re-equilibration with isocratic 0% B for 10 min. The smart parameter settings (SPS) were set for the target mass 700 m/z and for compound stability 100 %. The full MS scan mode was standard-enhanced (m/z 100 to 2000). The most abundant ion detected in each MS scan was selected for collision-induced dissociation (MS/MS) with 1.4 V collision energy. Ions of an m/z value in the ranges m/z 703.7-704.7 and m/z 771.4-772.4 were preferentially selected. This setup was used for an initial identification of the glycans. Afterwards, a second analysis with the same LC-parameter settings and different MS-settings was performed. In two further runs the target values of m/z 772.4 or 704.2 respectively were isolated and fragmented. Raw spectrum data were then processed using DataAnalysis 4.1 software (Bruker Daltonics). The MS2 fragmentation spectra acquired for each glycan were integrated into one averaged spectrum. Fragmentation analysis was performed with the help of GlycoWorkbench (ver. 2.1) [21].

## Results

### Identification of sialoglycoproteins specific to exosomes

**Figure 1:**
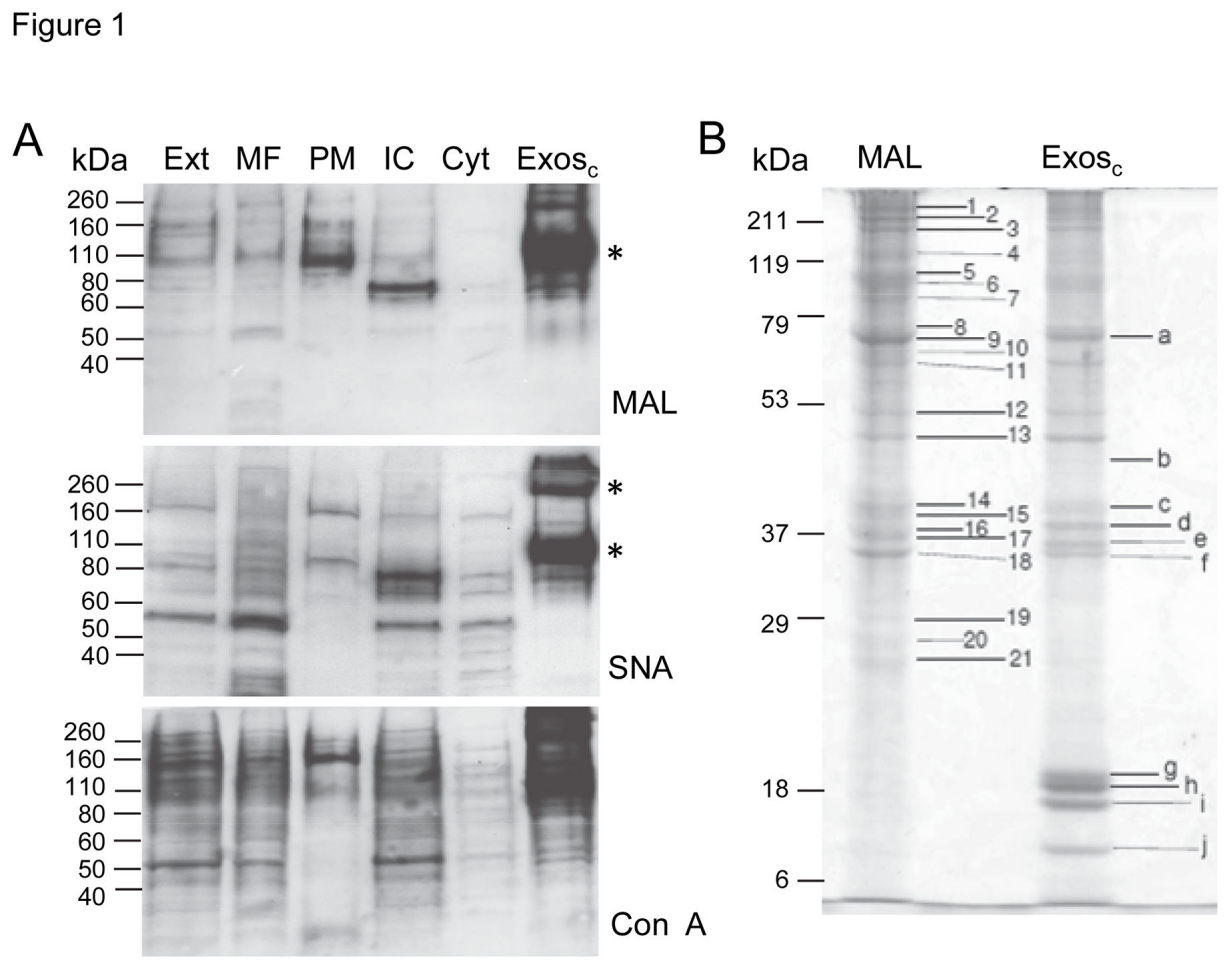
Glycoproteins from membrane fractions of SKOV3 cells. **A**. Lectin blotting of glycoproteins from cell extract (Ext), microsomal fraction (MF), plasma membrane (PM), intracellular fraction (IC) that consisted of non-biotinylated proteins, cytoplasmic fraction (Cyt) that consisted of the supernatant of the microsomal fraction and crude exosomes (Exos_c_) were analysed. Three μg total protein were applied per lane. Detection was performed using the chemiluminescent method. * Correspond to most abundant sialoglycoproteins enriched in the exosomes. Results were representative of at least two independent experiments. **B**. SDS-PAGE analysis of vesicle proteins from SKOV3 cells for peptide mass fingerprinting. A. MAL-binding glycoproteins (bands 1 to 21 identified in Table **1**) and total vesicles proteins (bands *a* to *j* were identified in Table **1**). Detection was with Coomassie G-250.

The microsomal fraction, plasma membrane and crude exosomes from SKOV3 cells have been analysed by lectin blotting with the lectins MAL (binds NeuAcα2,3Galβ1,4GlcNAc/Glc), SNA (binds NeuAcα2,6Gal/GalNAc) and Con A (binds α-mannosyl containing branched glycans predominantly of the high-mannose followed by hybrid- and biantennary complex type). The enriched microsomal fraction was obtained by differential centrifugation (100,000 x g pellet after clarification of the cell extract at 15,000 x g) [22], the plasma membrane enriched fraction was obtained after cell surface protein biotinylation followed by streptavidin-agarose affinity [15], and the crude exosomes consisted of the pellet of the cell supernatant centrifugation at 100,000x*g* (this fraction is enriched in exosomes and also contains other vesicles that are produced by the cells) [5,6]. MAL detected a major band at approximately 110 kDa in the crude exosomes, which was only faintly detected in the microsomal fraction or in the plasma membrane (Figure 1A, indicated with an asterisk). SNA detected two bands at apparent molecular mass of 260 kDa and between 80 and 110 kDa, which were almost not detected in the microsomal fraction nor in the plasma membrane (Figure **1A**, indicated with asterisks). Digestion with sialidases from *Vibrio cholerae*, *Artrobacter urefaciens* (cleave α2,3/6-linked sialic acid) and *Streptococcus pneumoniae* (cleaves α2,3-linked sialic acid) abolished MAL binding, whereas digestion with the former two but not the later abolished SNA binding thus corroborating the specificity of binding as previously reported [6]. Concanavalin A also showed a specific binding pattern for the crude exosomes. Incubation in the presence of 0.2 M methyl-α-D-mannopyranoside abolished detection corroborating the specificity of binding. These results showed that the glycosylation or glycoprotein profiles of crude exosomes were distinct from those of the microsomal fraction or plasma membrane. 

We then aimed at identifying the MAL-binding glycoprotein that was enriched in exosomes in view of its abundance and specificity. With that purpose solubilized proteins from crude exosomes were fractionated by MAL affinity chromatography (Figure **1B**). Marked bands *1* to *21* (Figure **1B**) have been excised and analysed by peptide mass fingerprinting after trypsin hydrolysis. For comparison bands *a* to *j* (Figure **1B**) from crude exosomes were also analysed by peptide mass fingerprinting (Table **1**). Identified proteins are shown in Table **1**. Several glycoproteins have been identified in the MAL eluate: bands 1, 2, 4-8, 10, 12. Cytoplasmic proteins (11,13–21) were also identified but they were due to non-specific binding to the resin since cytoplasmic glycoproteins do not contain sialic acid. 

**Table 1:** Identification of MAL-binding glycoproteins based on peptide mass fingerprinting.

**Band**	**Protein name**	**Accession number**	**Molecular mass**	**Matching peptides**	**Sequence coverage**	**ExoCarta**
MAL-lectin						
1	laminin, alpha 5, isoform CRA_c	gi|119595780	411358	24	7	+
2	laminin B2 chain	gi|186964	183195	14	9	+
3	unnamed protein product	gi|37138	133321	8	7	-
4	galectin-3-binding protein precursor	gi|5031863	66202	12	24	+
5	galectin-3-binding protein precursor	gi|5031863	66202	25	40	+
6	galectin-3-binding protein precursor	gi|5031863	66202	27	40	+
7	galectin-3-binding protein precursor	gi|5031863	66202	15	27	+
8	galectin-3-binding protein precursor	gi|5031863	66202	13	25	+
9	bovine albumin	gi|74267962	71186	52	53	+
10	galectin-3-binding protein precursor	gi|5031863	66202	7	12	+
11	pyruvate kinase	gi|35505	58411	7	16	+
12	cathepsin D preproprotein	gi|4503143	45037	16	40	+
13	mutant beta-actin (beta'-actin)	gi|28336	42128	13	31	+
14	annexin A2 isoform 2	gi|4757756	38808	19	58	+
15	annexin A1	gi|4502101	38918	8	27	+
16	L-lactate dehydrogenase A chain isoform 1	gi|5031857	36950	9	21	+
17	annexin A1	gi|4502101	38918	8	23	+
18	syntenin	gi|2795863	32568	11	30	+
19	proteasome subunit alpha type-2	gi|4506181	25996	6	34	+
20	prosome beta-subunit	gi|551547	25950	3	12	+
21	macropain subunit delta	gi|296734	19590	4	16	+
Exos_c_						
a	bovine albumin	gi|74267962	71186	51	57	+
b	histone macroH2A1.2	gi|3493529	39748	10	37	+
c	annexin A2	gi|56967118	38808	22	64	+
d	histone H1b	gi|356168	21721	13	38	+
e	histone H1.2	gi|4885375	21352	12	36	-
f	keratin, type II cytoskeletal 2 epidermal	gi|47132620	65678	14	25	-
g	histone H2B	gi|1568551	13928	15	81	-
h	histone H2B type 1-H	gi|4504269	13884	16	81	-
i	histone H2A type 1-C	gi|4504245	14097	12	53	+
j	nucleosome core particle	gi|347447327	11641	14	78	+

In the mass region of 100 kDa bands 4 to 8 and 10 (Table **1**) were identified as the galectin-3-binding protein (LGALS3BP). LGALS3BP has a predicted mass of 65,331 based on the amino acid composition, but its detection around 100 kDa indicated that it is heavily glycosylated, and, therefore, the different bands 4 to 8 and 10 may correspond to distinct glycoforms, which is in agreement with the detection by MAL-lectin blotting as a broad band. Furthermore, the presence of glycans can impair the binding of the Coomassie dye to the protein thus explaining that a stronger detection was not observed [23].

**Figure 2:**
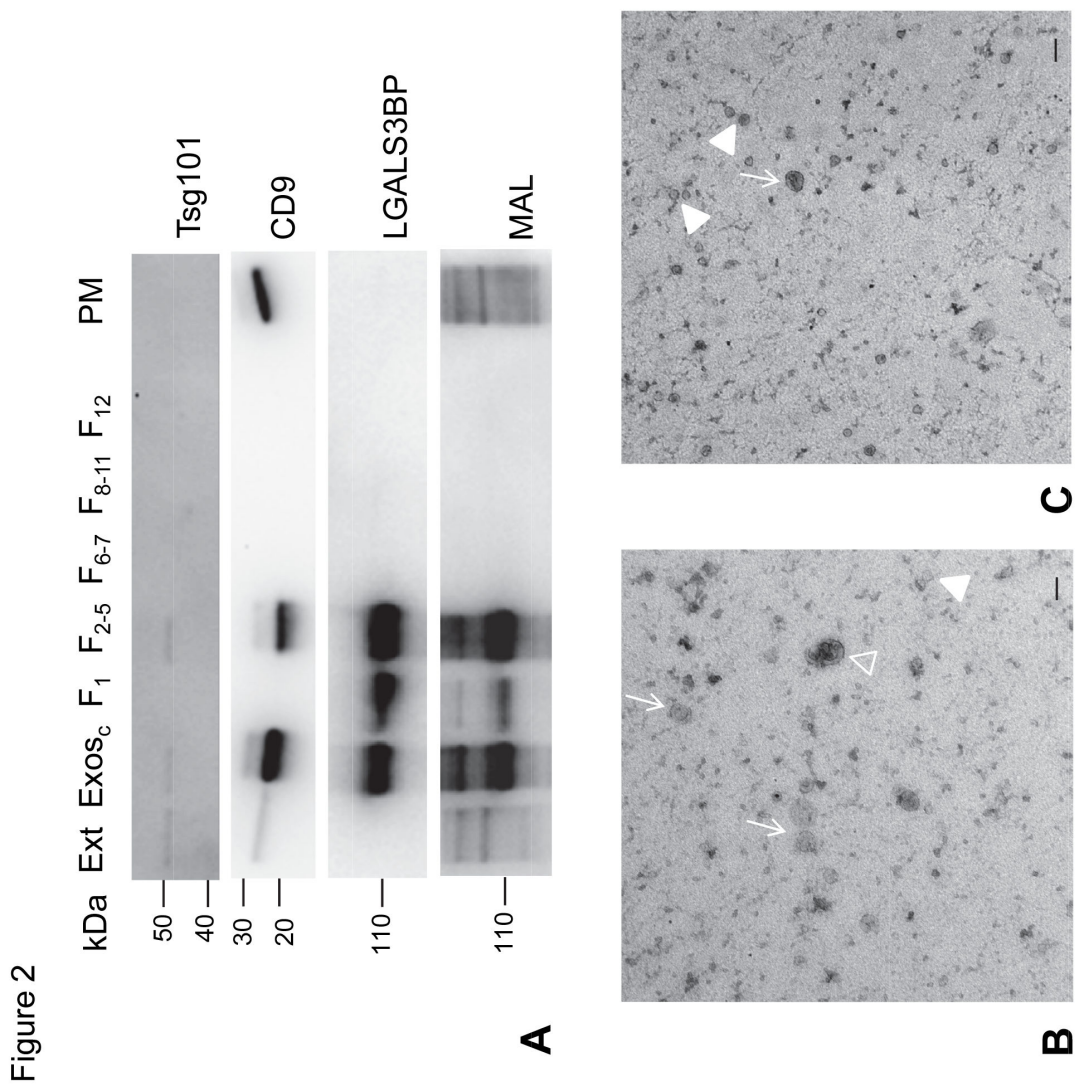
Characterization of exosomes obtained from SKOV3 cell supernatants. A) Immunoblotting of LGALS3BP in purified exosomes and plasma membrane. SKOV3 cell extracts (Ext), crude exosomes (Exos_c_), biotinylated plasma membrane proteins (PM), and fractions from the sucrose gradient used for Exos_c_ fractionation (F1, F2-5, purified exosomes, F6-7, F8-11 and F12). Three µg protein were applied per lane with the exception of F1, F6-7 and F12, where 20% of the amount obtained from the sucrose gradient was used. Detection was by the chemiluminescent method. Results were representative of two independent experiments. B) Electron microscopy visualization of crude exosomes (pellet collected after centrifugation at 100,000x*g* of pre-cleared supernatant). C) Electron microscopy visualization of purified exosomes (fractions 2 to 5 of sucrose gradient). Arrows, cup-shaped vesicles; open arrowhead, vesicles larger than 100 nm; closed arrowheads, vesicles approximately from 30 to 50 nm. The scale bar corresponds to 100 nm.

Crude exosomes were further purified by sucrose gradient as previously described [5,6] and fractions were analysed by immunoblot for the conventional exosome marker Tsg101 (tumor susceptibility gene 101; protein involved in the formation of multivesicular endosomes) [24], CD9 [6] and LGALS3BP (Figure **2**). Fractions 2-5 contained Tsg101, CD9 and also LGALS3BP (detected as a strong signal by immunoblot and MAL lectin blot). Since CD9 is also present at the plasma membrane and has recently been found in extracellular vesicles other than exosomes [24], biotinylated plasma membrane proteins were also analysed for comparison (Figure **2A**). As expected CD9 was detected at the plasma membrane contrary to Tsg101. Concerning LGALS3BP it was not detected at the plasma membrane which supported its specificity as an exosome marker.

Crude exosomes and purified exosomes were also visualized by electron microscopy. In agreement with the detection of biochemical markers, both preparations contained vesicles with exosome characteristics: cup-shaped vesicles up to approximately 100 nm (Figure **2 B,C**, arrows), and smaller vesicles between approximately 30 and 50 nm (Figure **2 B,C**, closed arrowheads). Larger vesicles or aggregates were also observed in crude exosomes and almost not detected in purified exosomes (Figure **2B**, opened arrowhead).

### N-glycosylation of glycoproteins from exosomes

**Figure 3:**
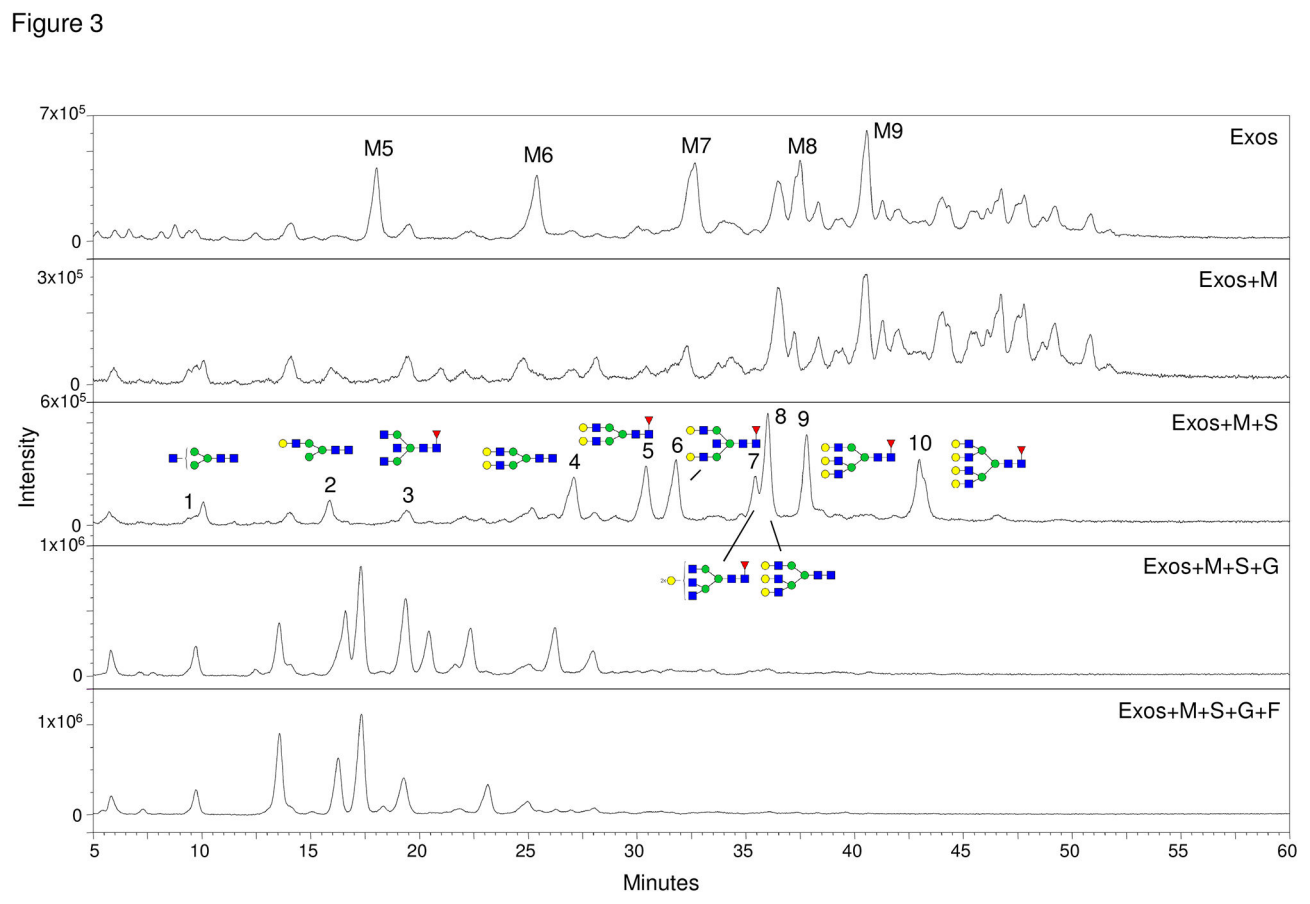
NP-HPLC analysis of 2-AB labeled N-glycans from purified exosomes. N-glycans were digested with mannosidase (M), sialidase (S), galactosidase (G) and fucosidase (F) as indicated. M5 to M9 consist of high mannose glycans Man_5_GlcNAc_2_ to Man_9_GlcNAc_2_, respectively. Structures of components from peaks 1 to 10 were identified by comparison with retention times of standards (Figure S1, Table S1) and after digestion with exoglycosidases.

N-Glycans have been released from glycoproteins of purified exosomes with peptide N-glycosidase F, fluorescently labeled with 2-aminobenzamide (2-AB) and analysed by NP-HPLC (Figure **3**). Peaks corresponding to high mannose glycans were found in exosomes and identified by their retention times in comparison with the reference standards (Figure **S1**, Table **S1**) and by their sensitivity toward mannosidase digestion (Figure **3**, second panel). Peaks corresponding to sialylated complex glycans were also found in exosomes, which corresponded to the peaks that disappeared after digestion with α2,3/6 specific sialidase (Figure **3**, third panel). Ten desialylated complex glycans (shown in Figure 3, third panel) have been identified by comparison with the retention times of reference oligosaccharide standards (Figure **S1**; Table **S1**) and considering the profiles obtained after digestion with exoglycosidases (Figure **3**). Complex glycans of the di-, tri- and tetraantennary type were found. Furthermore, diantennary glycans containing bisecting GlcNAc (peaks 3, 6) were also detected. Some of the structures were partially truncated (peaks 1, 2, 3), which could be due to the presence of glycosidases in the medium.

**Figure 4:**
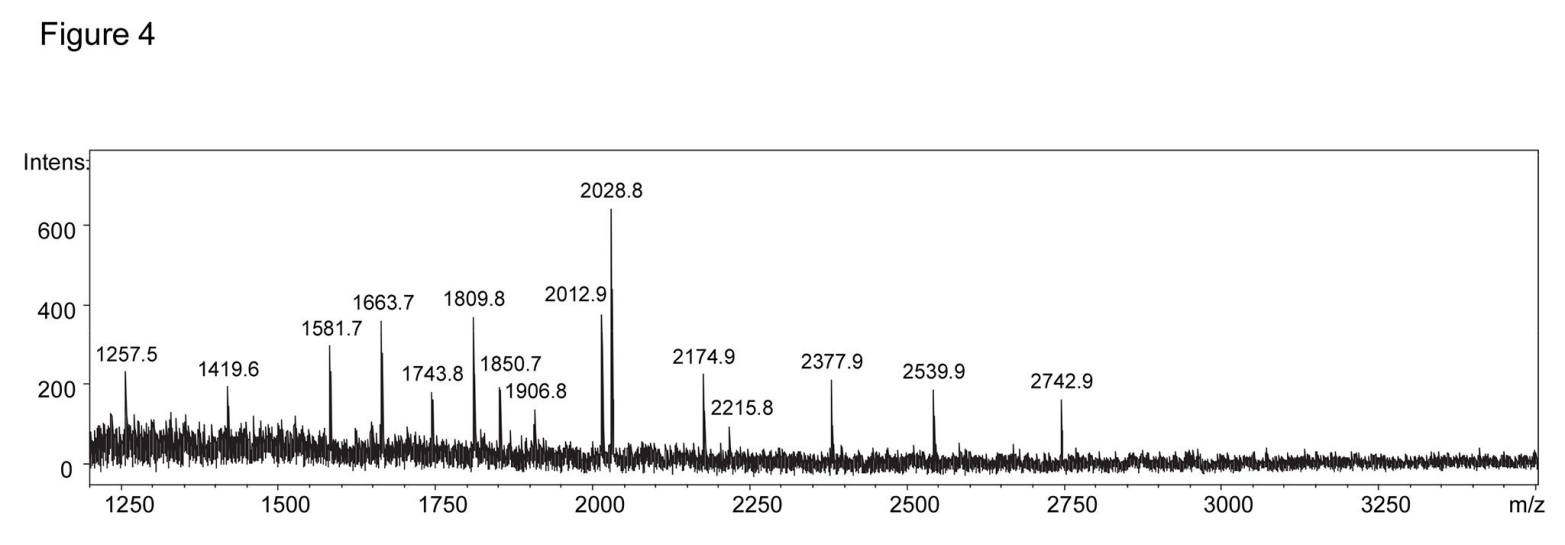
MALDI-TOF MS analysis of desialylated N-glycans from purified exosomes of SKOV3 cells. The *m/z* values of the first detected isotopic mass for major peaks are shown. Proposed compositions and compatible structures are presented in Table **2**.

Desialylated N-glycans have also been analysed by MALDI-TOF MS (Figure **4**). Based on the *m/z* values, the information from the NP-HPLC analysis/exoglycosidase digestion (Figure **3**) and the N-glycan biosynthetic pathway compatible glycan structures have been proposed for the major peaks (Table **2**). The exosomes contained complex glycans of the di-, tri- and tetraantennary type with or without proximal Fuc and high mannose glycans; structures lacking Gal residues have also been found (Table **2**). The presence of complex and high mannose glycans was also detected by MALDI-TOF MS in crude exosomes from another ovarian carcinoma cell line (OVM) (Figure **S2**). 

**Table 2:** Observed mass signals (*m/z*) by MALDI-TOF MS analysis and proposed composition of the *N*-glycans from exosomes.

**m/z**	**Proposed compositions**	**Compatible structures**	**NP-HPLC Peak**
1257.5 (1257.5)	Hex_5_HexNAc_2_	M5	M5
1419.6 (1419.5)	Hex_6_HexNAc_2_	M6	M6
1581.7 (1581.5)	Hex_7_HexNAc_2_	M7	M7
1646.6 (1647.6)	dHex_1_Hex_4_HexNAc_4_	FA2G1	-
1663.7 (1663.6)	Hex_5_HexNAc_4_	A2G2	4
1743.8 (1743.6)	Hex_8_HexNAc_2_	M8	M8
1809.8 (1809.7)	dHex_1_Hex_5_HexNAc_4_	FA2G2	5
1850.7 (1850.7)	dHex_1_Hex_4_HexNAc_5_	FA3G1, FA2BG1	-
1906.8 (1905.6)	Hex_9_HexNAc_2_	M9	M9
2012.9 (2012.7)	dHex_1_Hex_5_HexNAc_5_	FA3G2, FA2BG2	7, 6
2028.8 (2028.7)	Hex_6_HexNAc_5_	A3G3	8
2174.9 (2174.8)	dHex_1_Hex_6_HexNAc_5_	FA3G3	9
2215.8 (2215.8)	dHex_1_Hex_5_HexNAc_6_	FA4G2, FA3BG2	-
2377.9 (2377.9)	dHex_1_Hex_6_HexNAc_6_	FA4G3	-
2539.9 (2539.9)	dHex_1_Hex_7_HexNAc_6_	FA4G4	10
2742.9 (2743.0)	dHex_1_Hex_7_HexNAc_7_	FA4G4GlcNAc	-

Theoretical monoisotopic masses of the proposed glycan structures [M + Na]^+^ are shown in parenthesis. HexNAc: *N*-acetylhexosamine; dHex: deoxyhexose; Hex: hexose.

N-glycans have two core N-acetylglucosamine (GlcNAc); Mx, x represents the number of mannose (Man) on core GlcNAc; F at the start represents α1,6-linked core fucose (Fuc); A2, biantennary; A3, triantennary; A4, tetraantennary; B, bisecting GlcNAc; Gx, x represents number of galactose (Gal) [3];G1 or [6]G1 represents that Gal is on the α1,3 or α1,6 mannose; Sx (3,6), x represents number of sialic acids linked to Gal, the numbers in parentheses represent α2,3 or α2,6 linkage.

These mass signals have been detected from independent preparations of purified exosomes (spectrum shown in Figure 4) and crude exosomes (data not shown). Compatible structures considering the N-glycan biosynthetic pathway are shown. The structures identified by NP-HPLC (Figure 3) are indicated.

Peaks at *m/z* 2012.7 and *m/z* 2215.8 (detected at 2110.8 Da and 2313.9 Da, respectively, after derivatization with 2-AB) were analysed by LC-ESI Ion Trap MS and fragmentation by collision induced dissociation (CID). Fragments at m/z 910.25, 1038.29 and 1056.35 derived from the parent ion at 2012.7 identified a diantennary complex glycan with proximal Fuc and bisecting GlcNAc (Figure **S3**). Fragments at *m/z* 1442.28 and 1460.39 derived from the parent ion at 2215.8 identified a triantennary complex glycan with proximal Fuc and bisecting GlcNAc (Figure **S4**). A mass signal at *m/z* 2215.8 by MALDI-TOF MS was also found in crude exosomes from the OVM ovarian carcinoma cell line (Figure **S2**).

## Discussion

Exosomes are secreted vesicles that are produced from the fusion of multivesicular bodies with the plasma membrane and present specific biochemical and molecular characteristics. In the present work, we have identified the sialoglycoprotein LGALS3BP as an exosome marker from ovarian carcinoma cells and we also characterized the N-glycans from exosomes. 

LGALS3BP is a secreted glycoprotein that has been found in the extracellular matrix and in body fluids, in exosomes from cancer cells, urinary and plasma exosomes [25] and in prostasomes, which are exosome-like vesicles secreted by the prostate [26]. It has been found in several cancers where it has a negative prognostic value, it mediates cell adhesion [27] and it has immunostimulatory properties [28]. Exosomes interact with target cells and, therefore, they are responsible for the transmission of pathogenic proteins among cells in neurodegenerative diseases and also for the activation of the immune system [1]. Several mechanisms underlie the exosome/target cell recognition that include ligand-receptor interactions, with subsequent fusion of vesicles with the plasma membrane or internalization of whole vesicles via endocytic pathways [1,6]. Several proteins have been shown to be involved in the interaction, for example, integrins (beta1 and beta2) from B cell exosomes mediate adhesion to collagen-I, fibronectin, and tumor necrosis factor-alpha-activated fibroblasts [29], and the intercellular adhesion molecule 1 from exosomes mediates interaction with the lymphocyte function-associated antigen 1 underlying exosome capture in cells of the immune system [1]. To our knowledge LGALS3BP has not previously been described to be involved in exosome/target cell interaction, however, since it is known to bind several proteins that are found on the cell surface, such as, collagens IV, V, and VI, fibronectin and nidogen and galectin-3 [30] and integrin, beta1 [31], it is possible that due to its enrichment in exosomes it could play an important role in exosome/target cell interaction via components of the extracellular matrix with implications in exosome uptake. Further experiments are required to elucidate this matter. Supporting the presence of LGALS3BP in other cell lines was our previous observation of a strong MAL-binding glycoprotein at approximately 100 kDa in crude exosomes of ovarian carcinoma GG and m130 cells (data not shown), or H4 neuroglioma and HEK293 cells [6]. 

LGALS3BP has seven potential N-glycosylation sites which are occupied for truncated forms expressed in HEK293 cells [30]. The extensive N-glycosylation explains the broad band that we have observed around 110 kDa. The strong binding by the lectin MAL shows that it contains sialic acid and indicates that most of the glycans are of the complex-type. Further studies are required to elucidate if certain glycoforms are specifically enriched in the exosomes. 

Several other MAL-binding glycoproteins have also been identified in crude exosomes, such as laminin or cathepsin D, that had already been found by others in exosomes and are presented in the database Exocarta 2012 [25].

Concerning protein N-glycosylation in exosomes, the predominance of complex glycans has been found, with relatively high amounts of sialylation. This is in agreement with other reports where the enrichment of complex N-linked glycans into tumor microvesicles has been found [10]. However, exosomes also contained high mannose glycans, which is in agreement with the finding of this type of structures in urinary exovesicles [32] and in tumor microvesicles [10]. Initial results from our laboratory have indicated higher amounts of sialylated glycans by NP-HPLC in the exosomes relatively to plasma membrane or microsomal fraction (results not shown). Bisecting GlcNAc-containing N-glycans have been found in exosomes (Figure **3**, Figure **S3** and Figure **S4**). Accordingly, complex bisecting glycans have also been described in tissues of endometrioid ovarian cancer [33], and increased N-acetylglucosaminyltransferase III activity was found in human serum of hepatoma patients [34] and pancreatic carcinoma tissues [35]. However, in other types of cancer, for example, colorectal cancer, decreases in the levels of bisecting GlcNAc-containing structures have been observed [36]. Furthermore, the presence of bisecting GlcNAc has been associated with decreases in metastasis formation in several cancers [37]. In view of this, it will be necessary to further investigate the levels of bisecting GlcNAc in other ovarian cancer cell lines, in human tissues of different types and stages of ovarian cancer, and to study its specificity and functional role in the disease.

Exosomes have been used in nanoscale cancer vaccines for different types of cancer and although the results are promising, further studies need to be performed to engineer exosomes for cancer vaccine development [38]. Since glycosylation is a major hallmark of cancer where strong deregulation takes place one possible approach to improve the properties of exosomes as vaccines is to remodel their glycosylation, and in this context, knowing the detailed glycosylation properties of exosomes is of crucial importance. Furthermore, using LGALS3BP as a target to improve exosomes properties as vaccines also appears an appealing idea. 

In summary, we have identified sialoglycoproteins from the exosomes of ovarian carcinoma cells most relevant being LGALS3BP that constitutes an exosome marker sialoglycoprotein. Furthermore, the N-glycans from exosomes of ovarian carcinoma cells have been characterized in detail. The results open novel perspectives to explore the potential roles of N-glycans in exosome biology and as markers for ovarian cancer. 

## Supporting Information

Figure S1 **NP-HPLC analysis of 2-AB labeled reference oligosaccharide standards.** a, Man3GlcNAc2Fuc. b, Man5GlcNAc2 c, Man6GlcNAc2 d, Man7GlcNAc2 e, Man8GlcNAc2 f, Man9GlcNAc2 g, diantennary minus 2 Gal with proximal α1,6 Fuc. h, diantennary minus 2 Gal with bisecting GlcNAc with proximal α1,6 Fuc. i, diantennary minus 1 Gal with proximal α1,6 Fuc (C3 and C6). j, diantennary minus 1 Gal with bisecting GlcNAc with proximal α1,6 Fuc. k, diantennary with proximal α1,6 Fuc. l, diantennary with bisecting GlcNAc with proximal α1,6 Fuc. m, monosialylated (2,6) diantennary minus 1 Gal with proximal α1,6 Fuc (C3 and C6). n, monosialylated (2,6) diantennary with proximal α1,6 Fuc. o, disialylated (2,6) diantennary without proximal α1,6 Fuc. p, diantennary minus 2 Gal minus 1 GlcNAc without proximal α1,6 Fuc (C3). q, diantennary minus 2 Gal minus 1 GlcNAc with proximal α1,6 Fuc (C3). r, diantennary minus 2 Gal without proximal α1,6 Fuc. s, diantennary minus 1 Gal minus 1 GlcNAc without proximal α1,6 Fuc (C3). t, diantennary minus 1 Gal minus 1 GlcNAc with proximal α1,6 Fuc (C3). diantennary minus 1Gal without proximal α1,6 Fuc (C6). u, diantennary with proximal α1,6 Fuc. w, triantennary (2,4) with proximal α1,6 Fuc. v, triantennary (2,6) with proximal α1,6 Fuc. x, tetraantennary with proximal α1,6 Fuc. y, diantennary without proximal α1,6 Fuc. z, triantennary (2,4) without proximal α1,6 Fuc. aa, triantennary (2,6) without proximal α1,6 Fuc. ab, tetraantennary without proximal α1,6 Fuc. ac, triantennary (2,4) minus 3 Gal with proximal α1,6 Fuc. ad, triantennary (2,6) minus 3 Gal with proximal α1,6 Fuc. ae, tetraantennary minus 4 Gal with proximal α1,6 Fuc. af, triantennary (2,6) minus 3 Gal without proximal Fuc. ag, tetraantennary minus 4 Gal without proximal α1,6 Fuc. ah, triantennary minus 2 Gal with proximal Fuc. ai, triantennary minus 1 Gal with proximal Fuc. aj, triantennary minus 2 Gal without proximal α1,6 Fuc. ak, triantennary minus 1 Gal without proximal α1,6 Fuc
(PDF)

Figure S2 **MALDI/TOF-MS analysis of the total desialylated N-glycans from crude exosomes of ovarian carcinoma OVM cells.** Only one or two possible isomeric structures of selected peaks are presented. Graphical representations of glycans are consistent with the nomenclature of the Consortium for Functional Glycomics.
(PDF)

Figure S3 **LC-MS/MS structural analysis of the 2110.8 Da 2-AB labeled oligosaccharide.** The fragmentation spectrum of the triply charged ion species of a 2110.8 Da oligosaccharide is shown that could in principle relate to one (and more) of the structures presented in panel A) with the structural part specific for the bisecting GlcNAc motif highlighted. B) the annotated fragmentation spectrum. C) excerpts of the fragmentation spectrum with fragment ions that can only be generated by the bisecting GlcNAc motif. The first fragment has a mass of 910.25 Da which corresponds to two HexNAc and one Hex as well as a 2-AB label. The second fragment has one (m/z 1038.29) or two (m/z 1056.35) water remaining from the fragmentation of the glycosidic bonds and in addition to the fragment shown on the left has an additional deoxy-hexose.
(PDF)

Figure S4 **LC-MS/MS analysis of the 2313.9 Da 2-AB labeled oligosaccharide.** The fragmentation spectrum of the triply charged ion species of a 2313.9 Da oligosaccharide at m/z 772.0 is shown that could in principle relate to one (and more) of the structures presented in panel A) with the structural part specific for the bisecting GlcNAc motif highlighted. B) the annotated fragmentation spectrum. C) An excerpt of the fragmentation spectrum with fragment ions that can only be generated by the bisecting GlcNAc motif. The fragment has one (m/z 1442.28) or two (m/z 1460.39) water remaining from the fragmentation of the glycosidic bonds.
(PDF)

Table S1 **Structures of 2-AB labeled reference oligosaccharide standards shown in Figure S1.**
(PDF)
