# Supplementary figures and images for: Sialoglycoproteins and N-Glycans from Secreted Exosomes of Ovarian Carcinoma Cells
Source: PLoS One. 2013 Oct 24;8(10):e78631. doi: 10.1371/journal.pone.0078631 (PMC3840218; doi:10.1371/journal.pone.0078631)

Figure S1

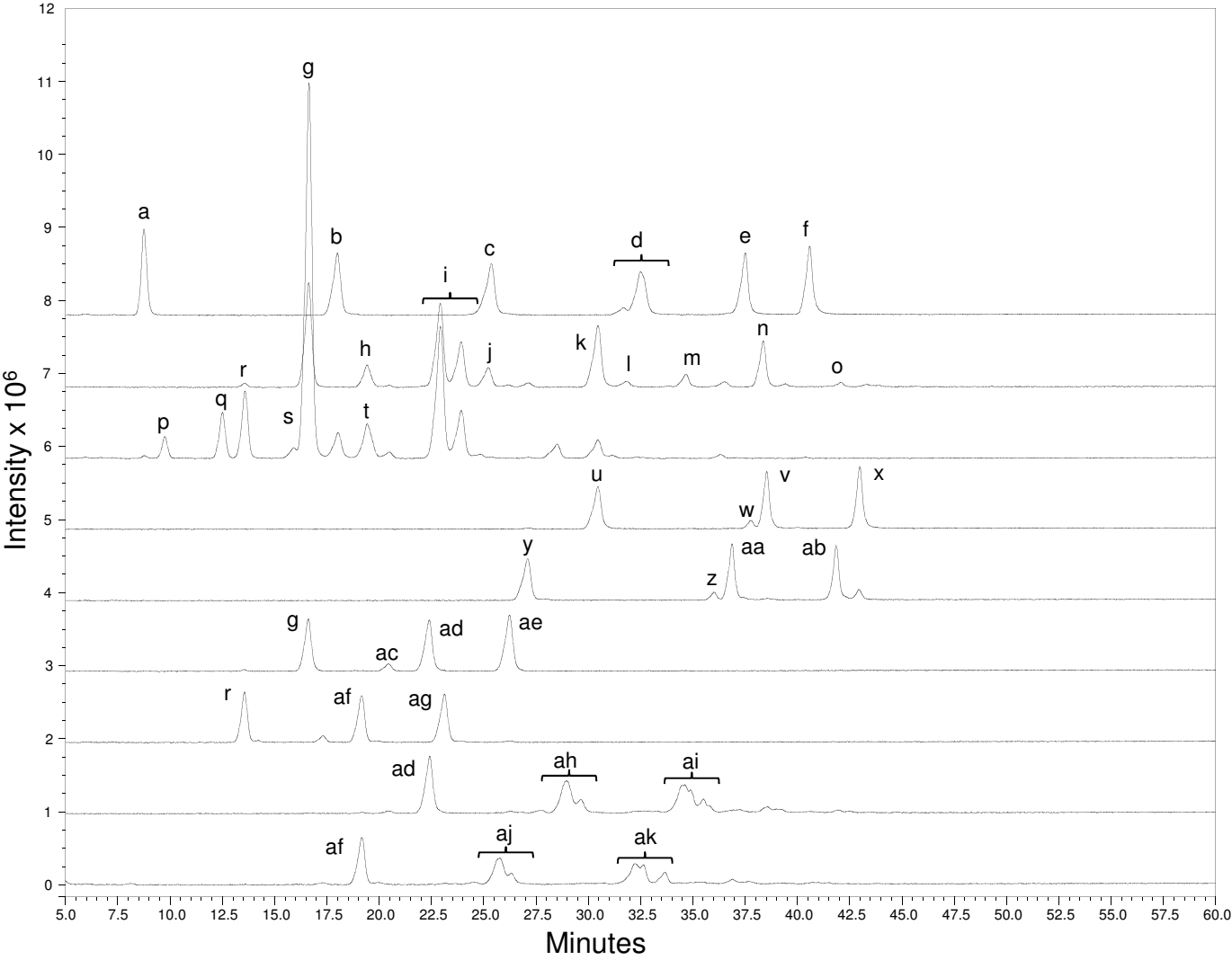

Supplement: Figure S1 — NP-HPLC analysis of 2-AB labeled reference oligosaccharide standards. a, Man3GlcNAc2Fuc. b, Man5GlcNAc2 c, Man6GlcNAc2 d, Man7GlcNAc2 e, Man8GlcNAc2 f, Man9GlcNAc2 g, diantennary minus 2 Gal with proximal α1,6 Fuc. h, diantennary minus 2 Gal with bisecting GlcNAc with proximal α1,6 Fuc. i, diantennary minus 1 Gal with proximal α1,6 Fuc (C3 and C6). j, diantennary minus 1 Gal with bisecting GlcNAc with proximal α1,6 Fuc. k, diantennary with proximal α1,6 Fuc. l, diantennary with bisecting GlcNAc with proximal α1,6 Fuc. m, monosialylated (2,6) diantennary minus 1 Gal with proximal α1,6 Fuc (C3 and C6). n, monosialylated (2,6) diantennary with proximal α1,6 Fuc. o, disialylated (2,6) diantennary without proximal α1,6 Fuc. p, diantennary minus 2 Gal minus 1 GlcNAc without proximal α1,6 Fuc (C3). q, diantennary minus 2 Gal minus 1 GlcNAc with proximal α1,6 Fuc (C3). r, diantennary minus 2 Gal without proximal α1,6 Fuc. s, diantennary minus 1 Gal minus 1 GlcNAc without proximal α1,6 Fuc (C3). t, diantennary minus 1 Gal minus 1 GlcNAc with proximal α1,6 Fuc (C3). diantennary minus 1Gal without proximal α1,6 Fuc (C6). u, diantennary with proximal α1,6 Fuc. w, triantennary (2,4) with proximal α1,6 Fuc. v, triantennary (2,6) with proximal α1,6 Fuc. x, tetraantennary with proximal α1,6 Fuc. y, diantennary without proximal α1,6 Fuc. z, triantennary (2,4) without proximal α1,6 Fuc. aa, triantennary (2,6) without proximal α1,6 Fuc. ab, tetraantennary without proximal α1,6 Fuc. ac, triantennary (2,4) minus 3 Gal with proximal α1,6 Fuc. ad, triantennary (2,6) minus 3 Gal with proximal α1,6 Fuc. ae, tetraantennary minus 4 Gal with proximal α1,6 Fuc. af, triantennary (2,6) minus 3 Gal without proximal Fuc. ag, tetraantennary minus 4 Gal without proximal α1,6 Fuc. ah, triantennary minus 2 Gal with proximal Fuc. ai, triantennary minus 1 Gal with proximal Fuc. aj, triantennary minus 2 Gal without proximal α1,6 Fuc. ak, triantennary minus 1 Gal without proximal α1,6 Fuc (PDF) [file pone.0078631.s001.pdf]

Figure S2

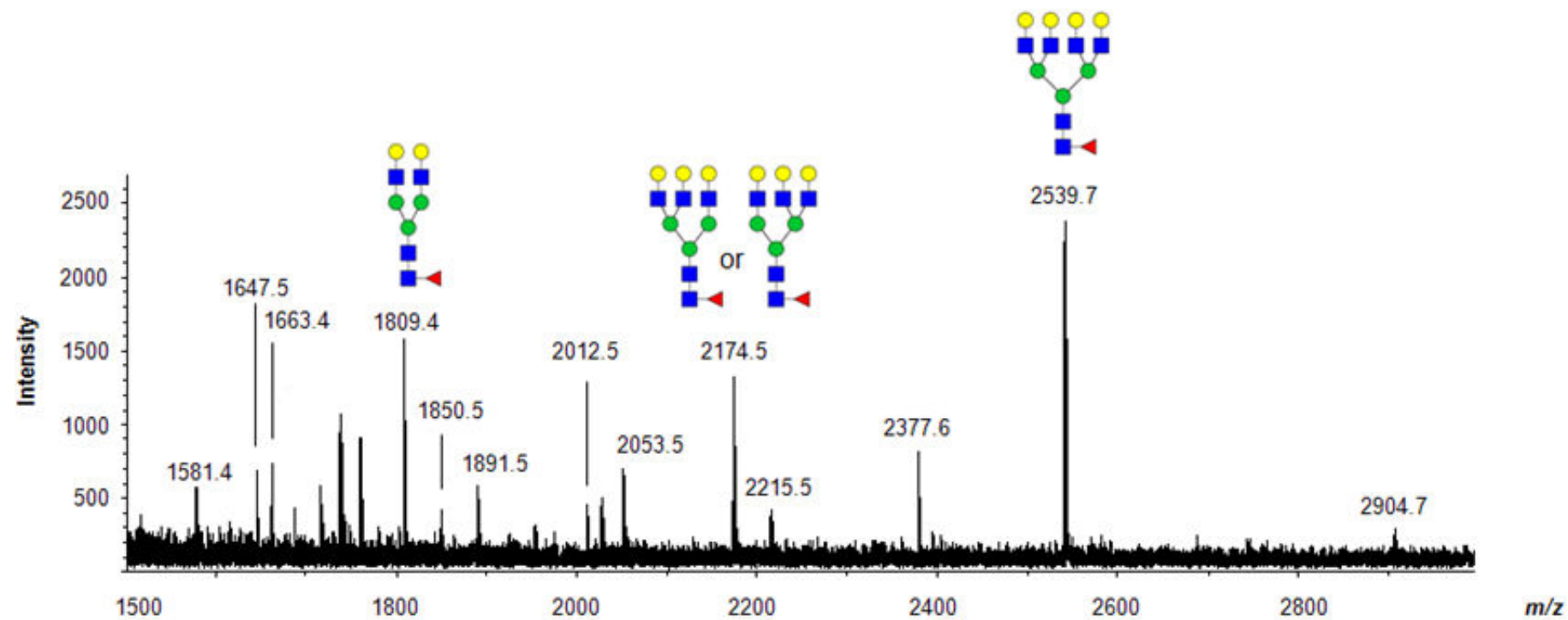

Supplement: Figure S2 — MALDI/TOF-MS analysis of the total desialylated N-glycans from crude exosomes of ovarian carcinoma OVM cells. Only one or two possible isomeric structures of selected peaks are presented. Graphical representations of glycans are consistent with the nomenclature of the Consortium for Functional Glycomics. (PDF) [file pone.0078631.s002.pdf]

Figure S3

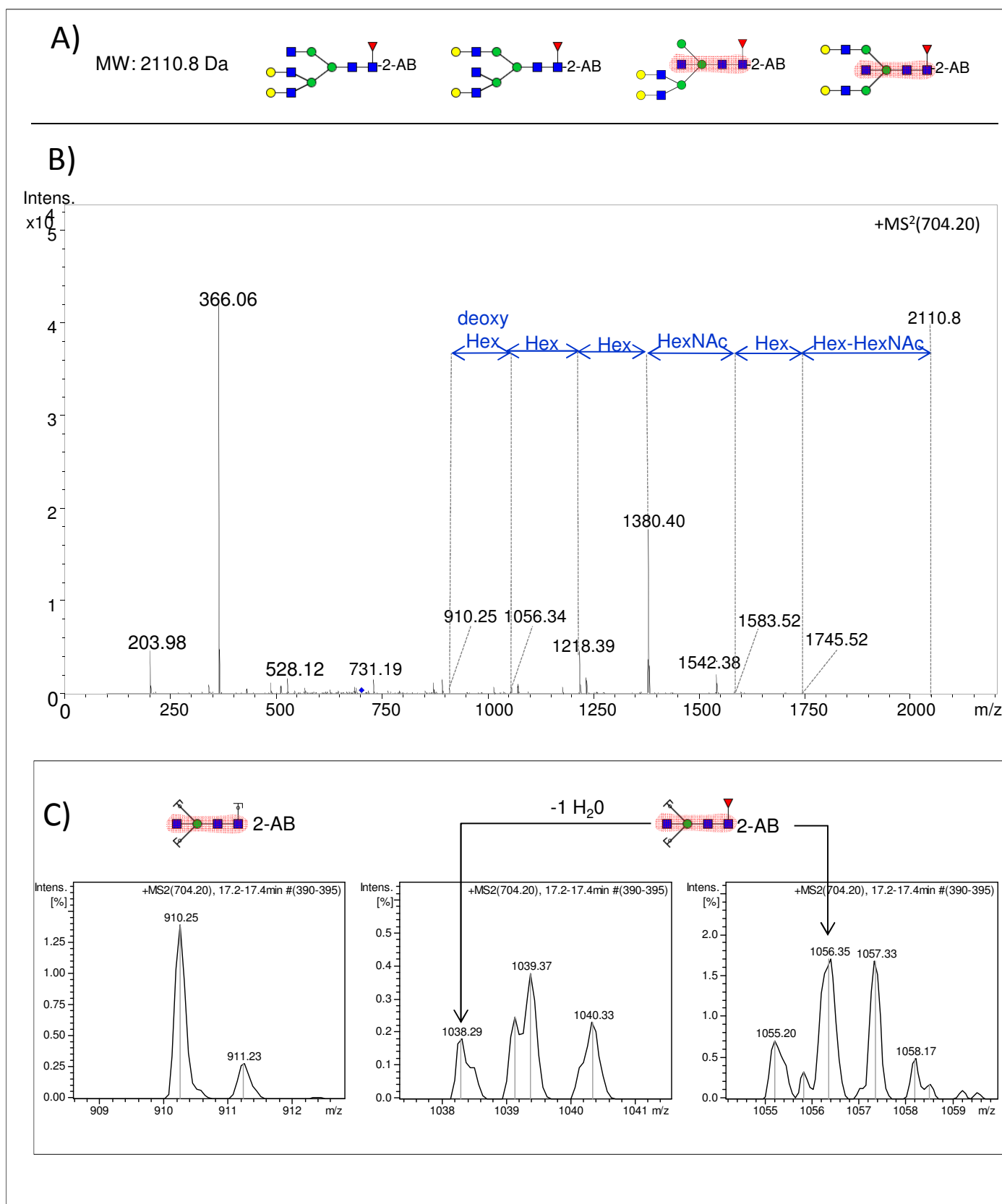

Supplement: Figure S3 — LC-MS/MS structural analysis of the 2110.8 Da 2-AB labeled oligosaccharide. The fragmentation spectrum of the triply charged ion species of a 2110.8 Da oligosaccharide is shown that could in principle relate to one (and more) of the structures presented in panel A) with the structural part specific for the bisecting GlcNAc motif highlighted. B) the annotated fragmentation spectrum. C) excerpts of the fragmentation spectrum with fragment ions that can only be generated by the bisecting GlcNAc motif. The first fragment has a mass of 910.25 Da which corresponds to two HexNAc and one Hex as well as a 2-AB label. The second fragment has one (m/z 1038.29) or two (m/z 1056.35) water remaining from the fragmentation of the glycosidic bonds and in addition to the fragment shown on the left has an additional deoxy-hexose. (PDF) [file pone.0078631.s003.pdf]

Figure S4

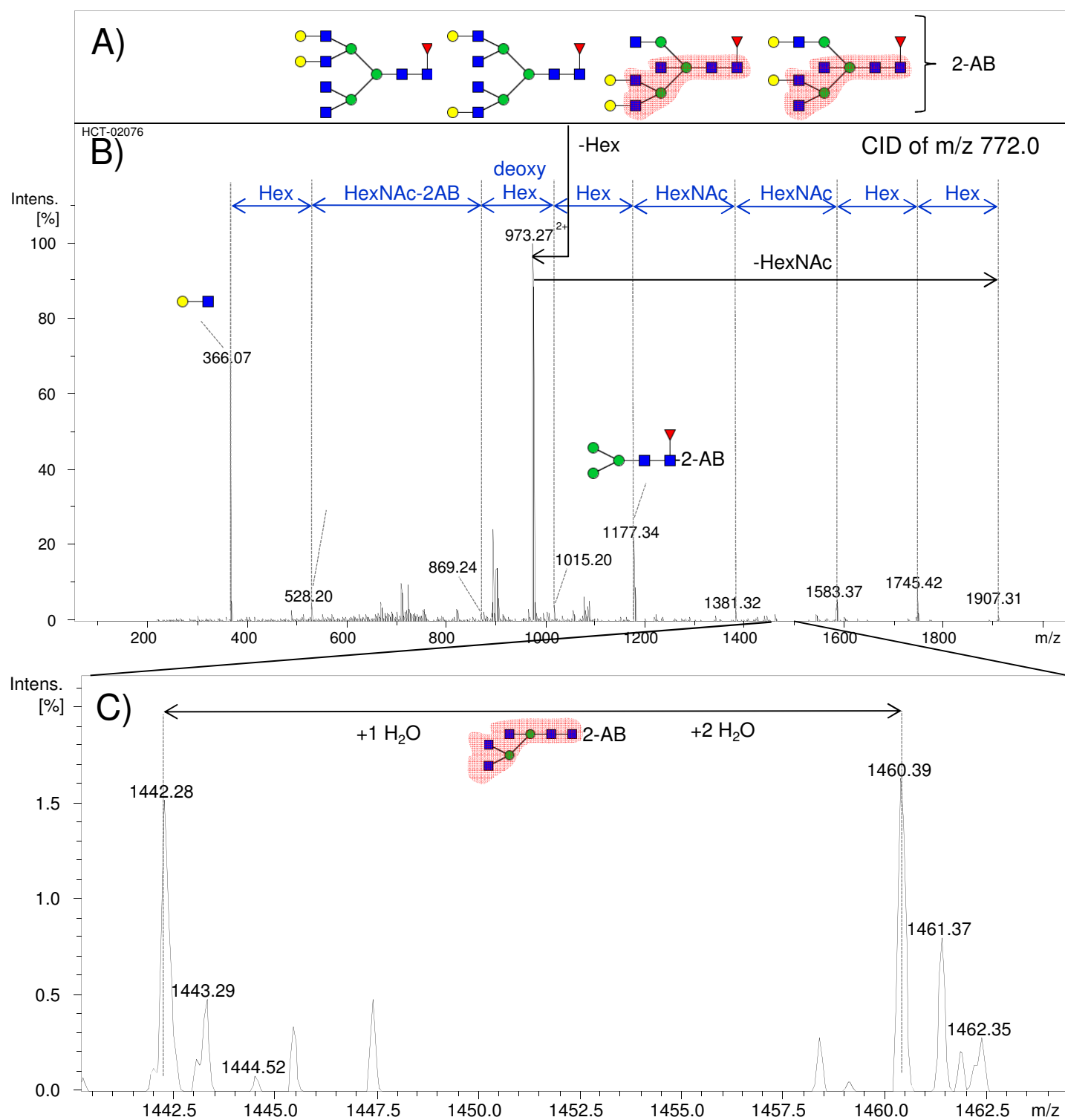

Supplement: Figure S4 — LC-MS/MS analysis of the 2313.9 Da 2-AB labeled oligosaccharide. The fragmentation spectrum of the triply charged ion species of a 2313.9 Da oligosaccharide at m/z 772.0 is shown that could in principle relate to one (and more) of the structures presented in panel A) with the structural part specific for the bisecting GlcNAc motif highlighted. B) the annotated fragmentation spectrum. C) An excerpt of the fragmentation spectrum with fragment ions that can only be generated by the bisecting GlcNAc motif. The fragment has one (m/z 1442.28) or two (m/z 1460.39) water remaining from the fragmentation of the glycosidic bonds. (PDF) [file pone.0078631.s004.pdf]
