# Supplementary material for: Sialoglycoproteins and N-Glycans from Secreted Exosomes of Ovarian Carcinoma Cells
Source: PLoS One. 2013 Oct 24;8(10):e78631. doi: 10.1371/journal.pone.0078631 (PMC3840218; doi:10.1371/journal.pone.0078631)
Supplement: Table S1 — Structures of 2-AB labeled reference oligosaccharide standards shown in Figure S1. (PDF) [file pone.0078631.s005.pdf]

Table S1. Structures of 2-AB labeled reference oligosaccharide standards shown in Figure S1.

**Peak**	**Designation**	**Structure (CFG)**	**Structure**
**a**	$Man_3GlcNAc_2Fuc$	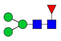	FM3
**b**	$Man_5GlcNAc_2$	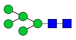	M5
**c**	$Man_6GlcNAc_2$	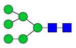	M6
**d**	$Man_7GlcNAc_2$	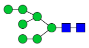	M7
**e**	$Man_8GlcNAc_2$	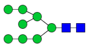	M8
**f**	$Man_9GlcNAc_2$	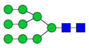	M9
**g**	Diantennary minus 2 Gal with proximal α1,6 Fuc	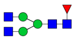	FA2
**h**	diantennary minus 2 Gal with bisecting GlcNAc with proximal α1,6 Fuc	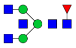	FA2B
**i**	diantennary minus 1 Gal with proximal α1,6 Fuc (C3 and C6)	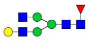<br>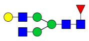	FA2[3]G1<br>FA2[6]G1
**j**	diantennary minus 1 Gal with bisecting GlcNAc with proximal α1,6 Fuc	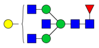	FA2BG1
**k**	diantennary with proximal α1,6 Fuc	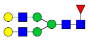	FA2G2
**l**	diantennary with bisecting GlcNAc with proximal α1,6 Fuc	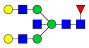	FA2BG2
**m**	monosialylated (2,6) diantennary minus 1 Gal with proximal α1,6 Fuc (C3 and C6)	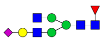<br>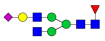	FA2[3]G1S1(6)<br>FA2[6]G1S1(6)
**n**	monosialylated (2,6) diantennary with proximal α1,6 Fuc	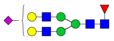	FA2G2S1(6)
**o**	disialylated (2,6) diantennary without proximal α1,6 Fuc	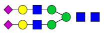	A2G2S2(6)
**p**	diantennary minus 2 Gal minus 1 GlcNAc without proximal α1,6 Fuc (C3)	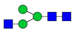	M3[3]GlcNAc
**q**	diantennary minus 2 Gal minus 1 GlcNAc with proximal α1,6 Fuc (C3)	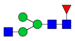	FM3[3]GlcNAc
**r**	diantennary minus 2 Gal without proximal α1,6 Fuc	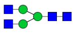	A2
**s**	diantennary minus 1 Gal minus 1 GlcNAc without proximal α1,6 Fuc (C3)	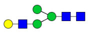	M3[3]Lac1
**t**	diantennary minus 1 Gal minus 1 GlcNAc with proximal α1,6 Fuc (C3)<br>diantennary minus 1Gal without proximal α1,6 Fuc (C6)	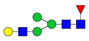<br>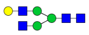	FM3[3]Lac<br>A2[6]G1
**u**	diantennary with proximal α1,6 Fuc	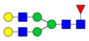	FA2G2

**w**	triantennary (2,4) with proximal α1,6 Fuc	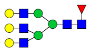	FA3G3
**v**	triantennary (2,6) with proximal α1,6 Fuc	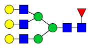	FA3G3
**x**	tetraantennary with proximal α1,6 Fuc	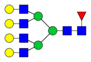	FA4G4
**y**	diantennary without proximal α1,6 Fuc	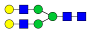	A2G2
**Z**	triantennary (2,4) without proximal α1,6 Fuc	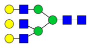	A3G3
**aa**	triantennary (2,6) without proximal α1,6 Fuc	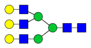	A3G3
**ab**	tetraantennary without proximal α1,6 Fuc	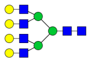	A4G4
**ac**	triantennary (2,4) minus 3 Gal with proximal α1,6 Fuc	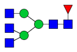	FA3
**ad**	triantennary (2,6) minus 3 Gal with proximal α1,6 Fuc	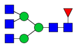	FA3
**ae**	tetraantennary minus 4 Gal with proximal α1,6 Fuc	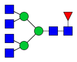	FA4
**af**	triantennary (2,6) minus 3 Gal without proximal Fuc	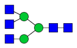	A3
**ag**	tetraantennary minus 4 Gal without proximal α1,6 Fuc	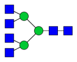	A4
**ah**	triantennary minus 2 Gal with proximal Fuc	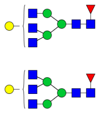	FA3G1
**ai**	triantennary minus 1 Gal with proximal Fuc	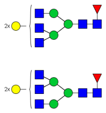	FA3G2
**aj**	triantennary minus 2 Gal without proximal α1,6 Fuc	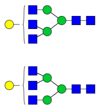	A3G1
**ak**	triantennary minus 1 Gal without proximal α1,6 Fuc	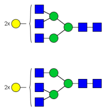	A3G2

Coloured structures are according to the representation of the Consortium of Functional Glycomics (CFG).
N-glycans have two core N-acetylglucosamine (GlcNAc); Mx, x represents the number of mannose (Man) on core GlcNAc; F at the start represents α1,6-linked core fucose (Fuc); A2, biantennary; A3, triantennary; A4, tetraantennary; B, bisecting GlcNAc; Gx, x represents number of galactose (Gal); [3]G1 or [6]G1 represents that Gal is on the α1,3 or α1,6 mannose; Sx (3,6), x represents number of sialic acids linked to Gal, the numbers in parentheses represent α2,3 or α2,6 linkage; Lac represents N-acetylactosamine.
